# Baicalin Inhibits Ferroptosis in Intracerebral Hemorrhage

**DOI:** 10.3389/fphar.2021.629379

**Published:** 2021-03-19

**Authors:** Lining Duan, Ying Zhang, Yuna Yang, Shiyu Su, Ligui Zhou, Po-Chieh Lo, Jiaying Cai, Yiqi Qiao, Min Li, Shuiqing Huang, Hong Wang, Yousheng Mo, Qi Wang

**Affiliations:** ^1^ Science and Technology Innovation Center, Guangzhou University of Chinese Medicine, Guangzhou, China; ^2^ Clinical Medical College of Acupuncture Moxibustion and Rehabilitation, Guangzhou University of Chinese Medicine, Guangzhou, China; ^3^ Laboratory Animal Center, Guangzhou University of Chinese Medicine, Guangzhou, China

**Keywords:** baicalin, Chinese herbal medicine, intracerebral hemorrhage, ferroptosis, hemin

## Abstract

Intracerebral hemorrhage (ICH) is a subtype of stroke characterized by high mortality and disability rates. To date, the exact etiology of ICH-induced brain injury is still unclear. Moreover, there is no effective treatment to delay or prevent disease progression currently. Increasing evidence suggests that ferroptosis plays a dominant role in the pathogenesis of ICH injury. Baicalin is a main active ingredient of Chinese herbal medicine *Scutellaria baicalensis*. It has been reported to exhibit neuroprotective effects against ICH-induced brain injury as well as reduce iron deposition in multiple tissues. Therefore, in this study, we focused on the protective mechanisms of baicalin against ferroptosis caused by ICH using a hemin-induced *in vitro* model and a Type IV collagenase-induced *in vivo* model. Our results revealed that baicalin enhanced cell viability and suppressed ferroptosis in rat pheochromocytoma PC12 cells treated with hemin, erastin and RSL3. Importantly, baicalin showed anti-ferroptosis effect on primary cortical neurons (PCN). Furthermore, baicalin alleviated motor deficits and brain injury in ICH model mice through inhibiting ferroptosis. Additionally, baicalin existed no obvious toxicity towards the liver and kidney of mice. Evidently, ferroptosis is a key pathological feature of ICH and baicalin can prevent the development of ferroptosis in ICH. As such, baicalin is a potential therapeutic drug for ICH treatment.

## Introduction

Intracerebral hemorrhage (ICH) is a common type of stroke characterized by high mortality and disability rates (Lim et al., 2020). Up to 58% of ICH patients die within 1 year after diagnosis and almost two-thirds of the survivors suffer from severe neurological deficits (Weimar and Kleine-Borgmann, 2017). ICH causes brain injury by primary physical disruption of the cellular architecture and secondary brain injuries such as edema, toxicity from metabolites of hemoglobin degradation and cell death (Duan et al., 2016; An et al., 2017). Currently, surgical evacuation of hematoma is the primary treatment of ICH. However, it has many limitations and may lead to secondary injuries (de Oliveira Manoel, 2020). Unfortunately, the curative efficacy of drug therapy is still not sufficient (Bower et al., 2019). Although the exact cause of ICH-induced brain injury is still unclear, increasing evidence suggests that the disorder of iron metabolism plays an important role in the pathogenesis of ICH injury (Wan et al., 2019).

Hemoglobin is degraded into iron, carbon monoxide and biliverdin by heme oxygenase (HO-1) following ICH injury. Large amounts of iron are thus released into the extracellular space (Zheng et al., 2015). Iron ions are subsequently released into the brain tissues 24h after hemorrhage. The levels of iron remain high for at least 28 days (Xiong et al., 2014; Zille et al., 2017). Accumulating evidence demonstrates that iron overload in ICH injury could be a potent contributor to perihematomal edema, peroxide accumulation and cell death (Garton et al., 2016; Wu et al., 2019). Ferroptosis is a newly identified form of programmed cell death characterized by the iron-dependent accumulation of lipid hydroperoxides (Dixon et al., 2012; Gao et al., 2019). The biochemical characteristics of ferroptosis include glutathione (GSH) antioxidant dysfunction, glutathione peroxidase 4 (GPX4) depletion and lipid peroxide accumulation. Cytotoxic compounds accumulation could lead to protein collapse, lipid destruction and death of neurons once lipid peroxide overwhelms the cellular antioxidant activity. This plays a key role in the pathogenesis of ICH (Zhang et al., 2018; Mao et al., 2019). Recent studies revealed that ferroptosis inhibitors have neuroprotective effects on ICH-induced second brain injury. For instance, it was reported that Ferrostatin-1 could attenuate neurological defects through reducing iron deposition in the perihematomal brain tissues (Chen et al., 2019). These findings strongly suggest that the interventions targeting ferroptosis could be potential strategies in the treatment of ICH-induced brain injury.

**FIGURE 1:**
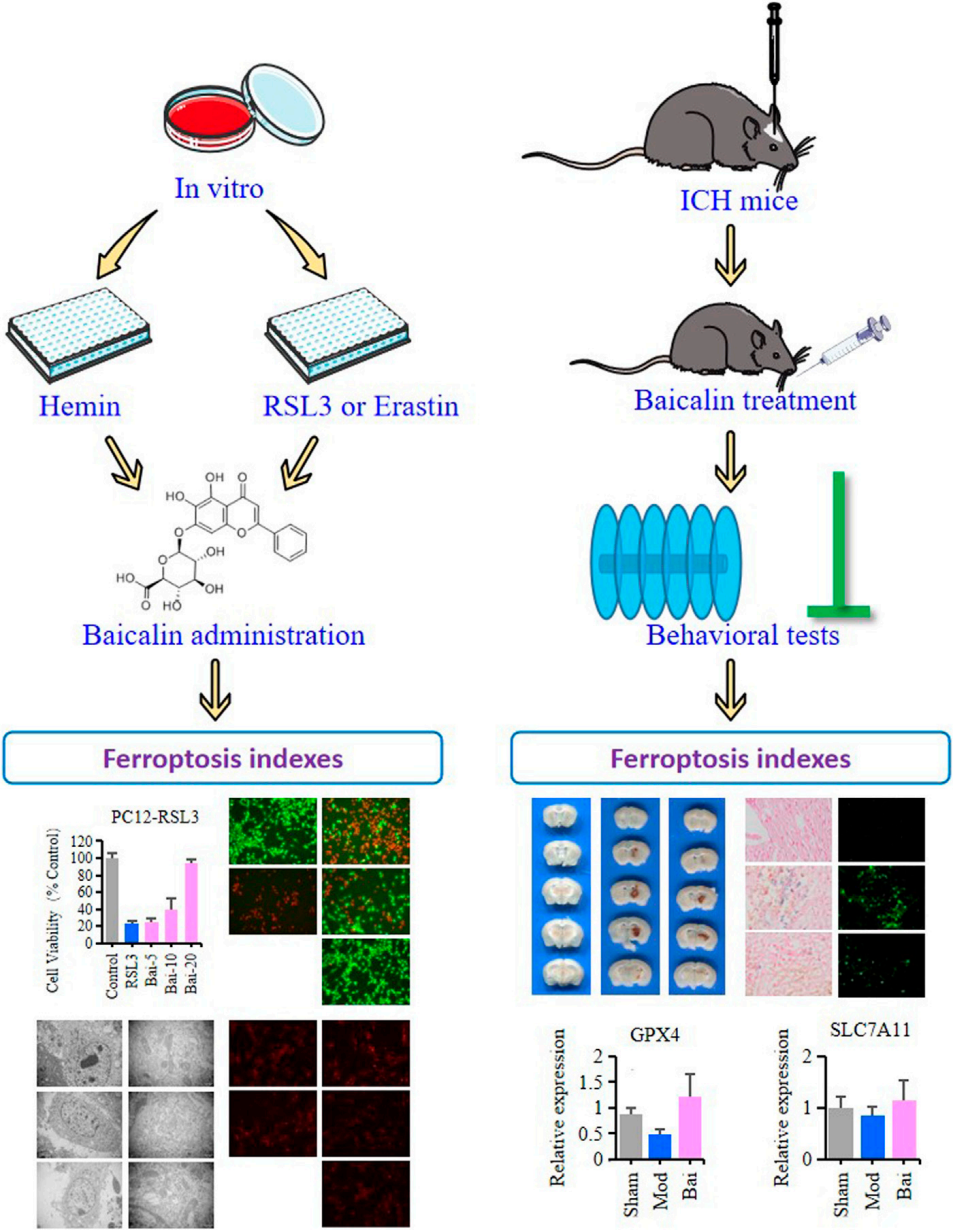
The graphical abstract of this study.

Baicalin is a main active ingredient of Chinese herbal medicine *Scutellaria baicalensis*. It possesses pharmacological properties such as antioxidant, antiapoptotic, and neuroprotective effects in various diseases (Chen et al., 2018; Fang et al., 2018; Zhou et al., 2019). Resent studies have demonstrated that baicalin can pass the blood-brain barrier (BBB) and ameliorate hemorrhagic brain injury effectively (Shi et al., 2017). Moreover, baicalin has been reported to reduce iron deposition in liver, kidney and brain tissues (Zhang et al., 2012; Guo et al., 2014). Based on these findings, we pointed out the hypothesis as follows: In ICH, baicalin might improve neural dysfunction through inhibiting ferroptosis in the brain tissues. Therefore, in this study, we seek to explore the protective mechanisms of baicalin against ferroptosis in ICH-induced brain injury (Figure 1).

## Materials and Methods

### Reagents and Materials

Baicalin was purchased from Herb purify Co., Ltd. (Chengdu, China). Rat pheochromocytoma PC12 cell line was obtained from American Type Culture Collection (Manassas,VA, United States). Dulbecco’s modified eagle medium (DMEM), fetal bovine serum (FBS) and Trypsin-ethylenediaminetetraacetic acid solution (Trypsin-EDTA) were purchased from Gibco (Carlsbad, CA, United States). Hemin, erasin and RSL3 were purchased from MedChemExpress (Monmouth Junction, NJ, United States). 3-(4, 5-dimethylthiazol-2-yl)-2, 5-diphenyltetrazolium bromide (MTT) solution was purchased from Merck KGaA (Darmstadt, Germany). Live/dead cell staining assay kit was obtained from KeyGene Biotech (Nanjing, China). The ROS fluorescent Probe-Dihydroethidium (DHE) kit was purchased from Biyuntian Biotechnology (Shanghai, China). Collagenase Type IV was obtained from the ThermoFisher Scientific (Springfield Township, NJ, United States). Prussian blue staining kit was procured from Servicebio (Wuhan, China). Fluoro-Jade B (FJB) staining kit was purchased from Merck KGaA (Darmstadt, Germany). Hematoxylin and eosin (HE) staining kit was purchased from Leagene Biotechnology (Beijing, China). The primary antibodies including rabbit monoclonal against GPX4 and rabbit monoclonal against solute carrier family 7 membrane 11 (SLC7A11) were obtained from Abcam, Inc(Cambridge, United Kingdom). The second antibodies including Goat anti-mouse, Goat anti-rabbit IgG, Alexa Fluor 488 anti-Rabbit IgG and Alexa Fluor 594 anti-Rabbit IgG were purchased form Cell Signaling Technology (Danvers, MA). Gutamic-oxaloacetic transaminase (GOT), alanine aminotransferase (GPT) and blood urea nitrogen (BUN) assay kits were obtained from the BiYunTian Biotech Company (Shanghai, China). Culture flasks (25 and 75cm^2^ growth area) and 96-well plates (0.32cm^2^ growth area per well) were purchased from Corning Costar Corp. (Cambridge, MA, United States). Ultrapure water was pretreated with the Milli-Q Plus System (Millipore Corporation, Bedford, MA, United States).

### Rat Pheochromocytoma PC12 Cells Cultures

PC12 cell line was cultured in DMEM supplemented with 10% FBS and 1% penicillin/streptomycin, and incubated at 37°C in an atmosphere of 5% CO_2_. The cells were then seeded in 96-well plates at a density of 5000 cells per well and detached using 0.25% Trypsin-EDTA after reaching 90% confluency for subculturing. The cells were then subjected to various tests and analyses after passaging for three generations.

### Primary Cortical Neurons Cultures

The primary cortical neurons (PCN) were prepared from the cerebral cortex of newborn (P0) C57BL/6 mice as described previously with minor modifications. Briefly, the brain tissues were collected and transferred to sterile Petri dishes containing dissection solution (HBSS on ice). The cortical tissues were isolated after careful separation of meninges and blood vessels under a stereomicroscope. Then the cortical tissues were washed by dissection solution 3 times and cut into pieces. After that, the cortical tissues were incubated with 0.125% Trypsin solution (Gibco, United States) containing 20U/ml DNase I (Sigma-Aldrich, United States) for 20 min in 37°C. The digestion was terminated by 10% fetal bovine serum. The cortical tissues were gently triturated with a flame-polished Pasteur pipette to procure a homogeneous cell suspension. After filtering using a 70μm cell strainer, the cell suspension was centrifuged at 800r/min for 5min. A total of 8 × 104cells/well were seeded into a 48-well plate coated with polylysine. After 2h, the medium was changed to fresh complete neurobasal medium consisting of Neurobasal-A medium (Gibco, United States), supplemented with 2% B27 (Thermo Scientific, United States), 1% penicillin-streptomycin (Gibco, United States) and 1X glutaMAX (Thermo Scientific, United States). The PCN were placed in a 37°C incubator and culture media were changed freshly every 3d. On days 5 after culture, the primary culture yielded mixed cells with neuronal, glia and non-neuronal were employed in this study.

### Cell Viability Assay

Cell viability was determined using the MTT assay kit. PC12 cells were cultured in 96-well plates for 24h in DMEM media and treated with hemin (80μM), RSL3 (0.5μM) or erastin (2.5μM) and corresponding concentrations of baicalin (0, 5, 10 and 20μM) to evaluate the cytoprotective effect of baicalin. The supernatant was then discarded, and 90μL DMEM and 10μL MTT solution were added to each well. This was followed by incubation of the cells in the dark for 4h after which the supernatant was discarded and 150μL of Dimethyl sulfoxide was added to each well. The cells were then agitated by shaking on an oscillator at low speed for 10min. There were six samples in each group for the MTT assay. The viability of the cells was then measured using a microplate reader (Countster, United States) at an absorbance of 490nm.

### Live/Dead Cell Staining Assay

Live/dead cell staining assay was conducted to determine the rate of apoptosis in PC12 cells treated with hemin (80μM), RSL3 (0.5μM) or erastin (2.5μM) and corresponding concentrations of baicalin (0, 5, 10 and 20μM) for 24h. The differentially treated cells were incubated with the staining working solution (2μM calcein acetoxymethyl ester, 8μM propidium iodide) for 30min in the dark and their images subsequently were viewed and captured using a fluorescent microscope (DMI8, Leica, Germany). There were four samples in each group for live/dead cell staining assay.

### Transmission Electron Microscope

The morphological features of mitochondria were observed under a JEM2000EX transmission electron microscope (TEM, Tokyo, Japan) to determine the effects of baicalin on the mitochondrial structure of PC12 cells treated with erastin and RSL3.

### Detection of ROS

To evaluate the effect of baicalin on ROS generation in PC12 cells, DHE kit was used to examine the ROS level in differentially treated PC12 cells. DHE is a cell-permeative dye that is oxidized to fluorescent ethidium bromide by superoxides and intercalates into DNA. Differentially treated PC12 cells were fixed with 4% PFA and then incubated with DHE for 30min in the dark at 37°C. The cells were then washed thrice to remove the extra DHE, and the ROS-positive cells were identified using a fluorescent microscope (DMI8, Leica, Germany). There were four samples in each group for DHE assay.

### Animals and Drug Administration

A total of 60 male C57BL/6 mice (10weeks old, 25–28g) were purchased from Guangzhou University of Chinese Medicine Experimental Animal Center (Guangzhou, China). The mice were maintained with enough food and water at 24°C, 60% relative humidity and 12/12h light/dark cycle. The mice were randomly divided into three groups: sham operation group (Sham), ICH model group (Mod) and baicalin group (Bai) (n = 20/group). Baicalin was suspended in 0.5% carboxymethylcellulose sodium solution. Given the extremely low solubility of baicalin, the concentration of baicalin solution was 0.5mg/ml. To achieve 20mg/kg/day dosage, the baicalin solution was administered to the mice in the Bai group by oral route twice at an interval of 1h within 2h after ICH injury onset. The remaining two groups received an equal volumes of saline through oral gavage. Since the second day after ICH, mice in the Bai group received 20mg/kg of baicalin solution while those in the remaining two groups received equal volumes of saline once a day for three consecutive days. The experiment was approved by the Laboratory Animal Ethics Committee of Guangzhou University of Chinese Medicine (Guangzhou, China) and performed in accordance with guiding principles of the United States National Institutes of Health for the care and use of laboratory animals.

### Establishment of Intracerebral Hemorrhage Mice Model

The ICH model was established as previously described (Gao et al., 2017). After anesthetization by intraperitoneal injection of 2% pentobarbital sodium (40mg/kg), the mice were kept in prone position in a stereotaxic frame (RWD,—China). A burr hole (1mm) was made using a dental drill (2.0mm lateral right of the bregma, and 3.5mm depth below the skull surface). Type IV collagenase (0.1U) dissolved in 1µL saline was injected into the hole at a rate of 0.2µL/min to induce ICH injury in the Mod and Bai groups. The drill was left in the position for 10min to prevent reflux and then withdrawn at a rate of 1mm/min. Mice in the Sham group received an equal amount of normal saline instead of collagenase. Then, the mice were placed on a heating pad (25°C) after surgery to prevent hypothermia during recovery. In this study, exclusion criteria of the *in vivo* experiment were listed as following: 1) The animals who died spontaneously during the ICH modeling surgery. 2) The animals who showed no limb hemiplegia after modeling.

### Behavioral Test

Behavioral tests were conducted after baicalin treatment. A number of 10–12 animals per group was used for the behavioral test. Rotarod test and pole test were used to assess motor deficits after ICH injury as previously described (Wang et al., 2018). In the rotarod test, the mice were placed on a rotating rod at a speed of 20 rounds per minute for 120s. The latency to fall of the mice was then recorded. It was defined as the time each mouse could maintain its balance on the apparatus. The number of drops within 120s was also recorded. Pole test was conducted to measure the coordinated function of limb movement and the sense of balance. The mice were placed on the top of a pole, which had a diameter of 0.9cm and a height of 50cm, and allowed to climb down thrice at 5min intervals without interference. The average time each mouse spent in turning and traveling down the pole was recorded.

### Hematoma Assessment

Hematoma assessment was conducted after sacrificing the mice. The brains of six mice in each group were quickly removed and cut into 1mm thick brain sections after perfusion with PBS. The sections were then imaged with an Epson Perfection V370 Photo scanner (Epson China, Beijing, China) and the hematoma volume of each section was analyzed using the Image-Pro Plus software. Hematoma volume in cubic millimeters was calculated as the mean of the summation of the hematoma areas multiplied by the interslice distance (1mm).

### Preparation of Brain Paraffin Slices

After the behavioral tests were completed, four mice in each group were anesthetized and then transcardially perfused with PBS followed by 4% PFA. The mice were decapitated post-perfusion and the whole brains, livers and kidneys were extracted and post-fixed in 4% PFA overnight at 4°C. The tissues were finally embedded in paraffin and cut into 5μM thick sections for Prussian blue, FJB, immunofluorescent staining and HE staining.

### Prussian Blue Staining

Prussian blue staining was utilized to reveal the iron-labeled cells of perihematoma brain tissues in blue. Paraffin-embedded sections were dewaxed using xylene and rehydrated with gradient ethanol. They were then stained with Perls Prussian blue stain for 15min. The sections were rinsed with distilled water to remove the excess Perls Prussian blue stain and then further stained with hematoxylin for 30s. After that, they were subjected to gradient ethanol dehydration, made transparent using dimethyl benzene, and then mounted with neutral resin cover slides. Finally, the sections were observed and imaged using a fluorescence microscope (DMI8, Leica, Germany).

### Fluoro-Jade B Staining

FJB staining was used to assess neuronal degradation in the perihematoma brain tissues. The brain sections were deparaffinized using xylene and rehydrated with gradient concentrations of ethanol. Then they were incubated in a 80% ethanol solution containing 1% sodium hydroxide for 5min. The sections were further incubated in 0.06% potassium permanganate for 20min and rinsed with distilled water. After that, they were stained with 0.0004% FJB solution for 30min and mounted with neutral gum after dehydration and induction of transparency. A total of three to four fields of view were randomly selected in each section to count the number of FJB positive cells under a fluorescence microscope (DMI8, Leica, Germany).

### Immunofluorescence Staining

The brain sections underwent antigen repair using 0.01M citrate buffer at 90°C for 30min. They were saturated and permeabilized in 0.1% Triton X-100 and blocked with the goat serum at room temperature. Then the sections were incubated with primary antibodies of anti-GPX4, or anti-SLC7A11 at 4°C overnight. Next, they were incubated with goat anti-mouse or goat anti-rabbit IgG at 37°C for 20min and stained with Alexa Fluor 488 anti-rabbit IgG or Alexa Fluor 594 anti-rabbit IgG at room temperature in the dark for 1h. Images were captured using a fluorescent microscope (DMI8, Leica, Germany) and the positive cells enumerated from three to four different views in each sample. There were four samples in each group for immunofluorescence staining.

### HE Staining

The paraffin sections of liver and kidney in four mice of each group were deparaffinized in xylene, and rehydrated with different concentrations of alcohol and distilled water. For HE staining, the sections were incubated in hematoxylin solution for 5 min and washed with tap water, then stained in an eosin solution for 1 min. Next, the sections were dehydrated with alcohol, cleared with xylene and mounted with neutral gum. After that, the sections were viewed and imaged with an optical microscope (model DMi8, Leica, Germany).

### Serum Biochemical Indicators Detection

Blood samples were taken from six mice in each group after behavioral tests. Plasma supernatants were collected following centrifugation at 900gx for 10min at 4°C and stored at – 80°C. The levels of serum GOT, GPT and BUN were analyzed using corresponding assay kits according to the instructions.

### Quantitative Real-Time Polymerase Chain Reaction

Total RNA was extracted from the perihematoma brain tissues of six mice in each group using the TRIzol reagent (Sigma, United States). Templates of cDNA were prepared using the PrimeScript™ RT Master Mix (Takara, Beijing, China) by reverse transcription. The templates were then diluted in the ratio of 1:3 and subjected to amplification on a real-time fluorescence quantitative PCR instrument (ABI, CA, United States). Amplification of each sample was repeated thrice and the average value was used to calculate the relative content of the product. Primers used are listed in Table 1. GAPDH was utilized as the housekeeping gene. The relative mRNA concentrations were measured by E = 2^−ΔΔCt^, and the key threshold cycle (CT) value was examined in each reaction.

**TABLE 1:** Sequences of primers designed for RT-qPCR.

Gene	Forward sequence	Reverse sequence
GPX4	GAT GGA GCC CAT TCC TGA ACC	CCC TGT ACT TAT CCA GGC AGA
SLCA11	GGC ACC GTC ATC GGA TCA G	CTC CAC AGG CAG ACC AGA AAA
SLC3A2	TGA TGA ATG CAC CCT TGT ACT TG	GCT CCC CAG TGA AAG TGG A
TFRC	GTT TCT GCC AGC CCC TTA TTA T	GCA AGG AAA GGA TAT GCA GCA
DMT1	CAA TGT CTT TGT CGT GTC CGT	GCG ACC ATT TTA GGT TCA GGA AT
GAPDH	GGT​TGT​CTC​CTG​CGA​CTT​CA	TGG​TCC​AGG​GTT​TCT​TAC​TCC

### Western Blot Analysis

The extracts of the perihematoma brain tissues of six mice in each group were obtained by lysis with RIPA buffer. Bicinchoninic acid (BCA) assay was employed to quantify the protein concentration. Samples were separated by SDS–polyacrylamide gel electrophoresis (SDS–PAGE) and transferred to polyvinylidene difluoride (PVDF) membranes (Millipore, Billerica, MA, United States), blocked by 5% BSA for 1.5h at room temperature. Then membranes were incubated with the primary antibodies anti-GPX4 and anti-SLC7A11 specific for target proteins overnight at 4°C, and then incubated with the secondary antibodies Alexa Fluor 488 anti-rabbit IgG or Alexa Fluor 594 anti-rabbit IgG for 1h at 37°C. Detection was performed using the Odyssey Infrared Imaging System (LI-COR Inc., United States) by a fluorescent readout and quantified by Bio-Rad Image Lab 5.2.1 software (Bio-Rad Laboratories, California, United States).

### Statistical Analysis

Experimental data were shown as mean ± standard deviation (SD) or mean ± standard error (SE), and they were analyzed by the Statistical Package for the Social Sciences software (SPSS; version 25.0). Comparison between two groups was performed using t tests. Comparisons between multiple groups were made using one way analysis of variance (ANOVA) followed by the least significant difference (LSD) test. The *p*-value < 0.05 indicated statistical significance.

## Results

### Baicalin Inhibited Hemin-Induced Ferroptosis in PC12 Cells

**FIGURE 2:**
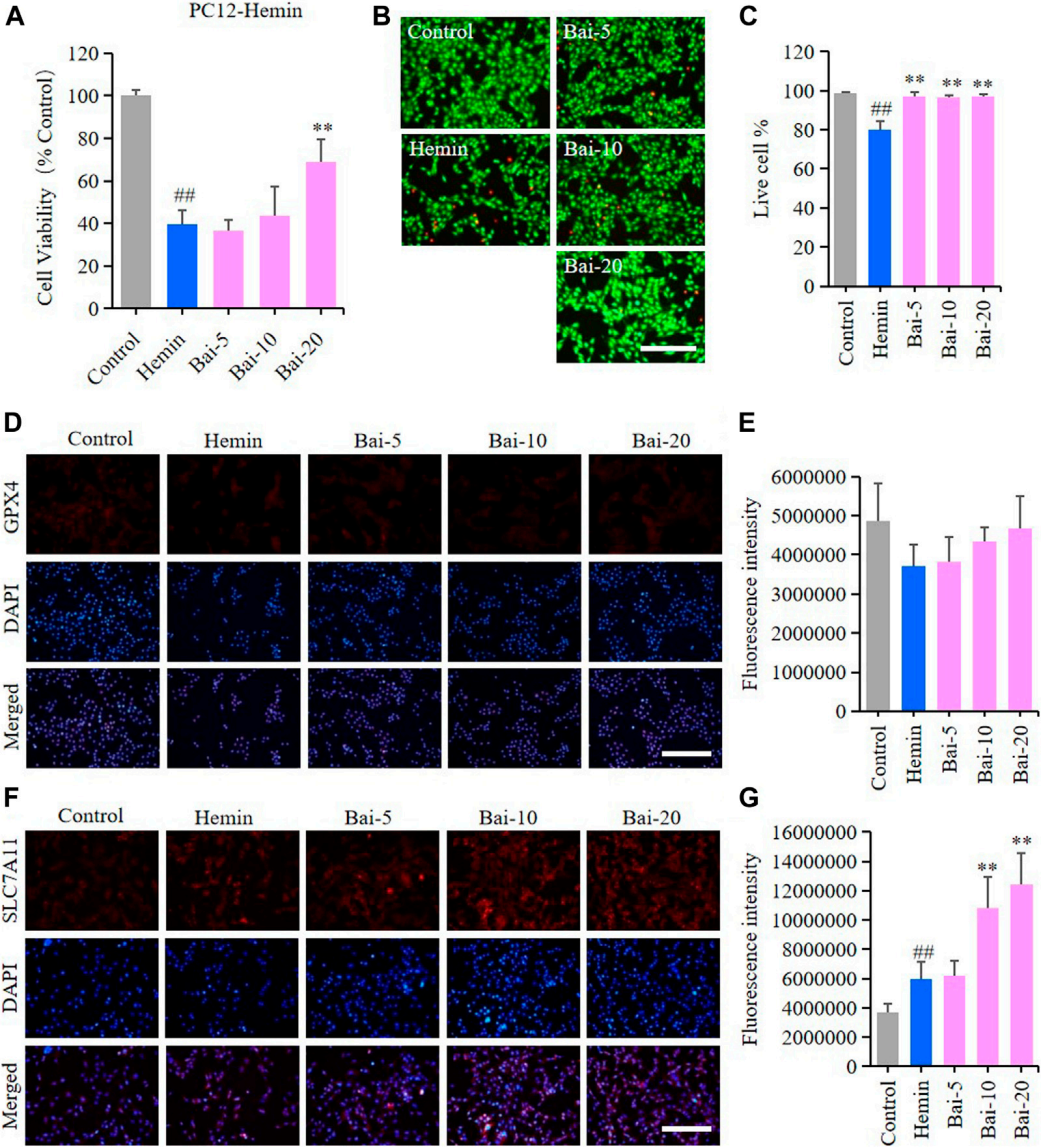
Effect of baicalin on hemin-induced ferroptosis in PC12 cells. PC12 cells were cultured in 96-well plates, treated with hemin (80μM) and corresponding concentrations of baicalin (0, 5, 10 and 20μM) for 24h. Effect of baicalin on the viability of PC12 cells was tested by MTT assay (*n* = 6) **(A)** and live/dead cell staining assay. Bar = 200μm **(B)**. **(C)** The ratio of living cells was counted in different groups (*n* = 4). **(D)** The expression of GPX4 was analyzed using immunofluorescence staining. Bar = 200μm. **(E)** Relative fluorescence intensity statistics of the expression of GPX4 in different groups (n = 4). **(F)** The expression of SLC7A11 was analyzed using immunofluorescence staining. Bar = 200μm. **(G)** Relative fluorescence intensity statistics of the expression of SLC7A11 in different groups (*n* = 4). The experiments were repeated at least three times with three replicates and data were expressed as mean ± SD. ^##^ *p* < 0.01 compared with the control group and **p < 0.01 compared with the hemin-treated group.

MTT assay and live/dead cell staining assay were performed to explore the effects of baicalin on hemin-induced cytotoxicity in PC12 cells. MTT assay results showed that hemin decreased the viability of PC12 cells, while baicalin enhanced cell viability in a concentration-dependent manner (Figure 2A). Likewise, the live/dead cell staining assay revealed that baicalin significantly reversed hemin-induced PC12 cell death (Figures 2B,C). GPX4 and SLC7A11 levels were further detected using immunofluorescence staining to confirm the protective effect of baicalin against ferroptosis in hemin-treated PC12 cells. Hemin caused a reduction in GPX4 level, while baicalin remarkably increased the GPX4 expression (Figures 2D,E). Furthermore, the GPX4 depletion induced by hemin lead to a compensatory increase of upstream protective regulator SLC7A11 expression, while baicalin treatment effectively enhanced the expression of SLC7A11 (Figures 2F,G). These results strongly suggested that baicalin could prevent hemin-induced cellular toxicity by inhibiting ferroptosis.

### Baicalin Inhibited RSL3-Induced Ferroptosis in PC12 Cells

**FIGURE 3:**
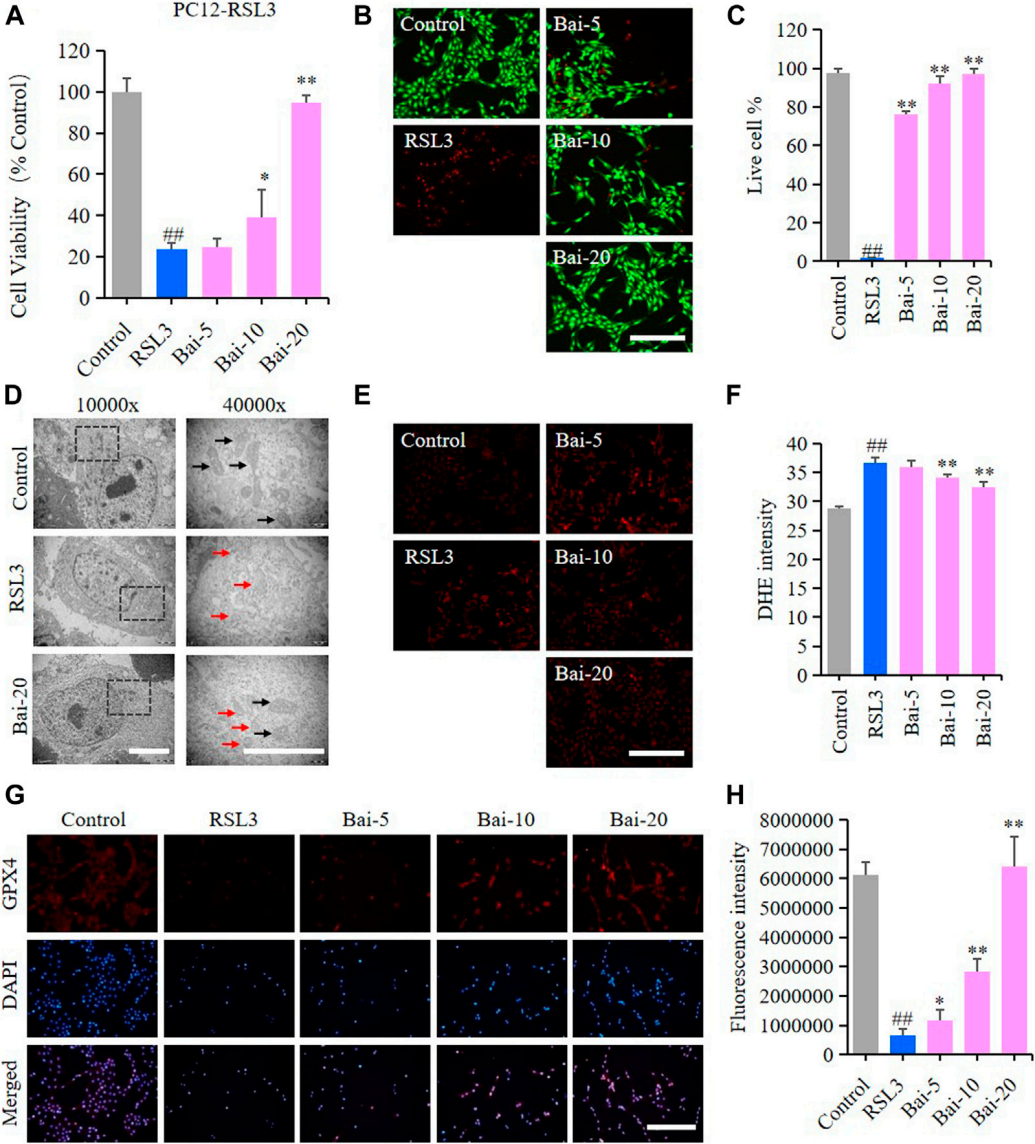
Effect of baicalin on RSL3-induced ferroptosis in PC12 Cells. PC12 cells were cultured in 96-well plates, treated with RSL3 (0.5μM) and corresponding concentrations of baicalin (0, 5, 10 and 20μM) for 24h. Effect of baicalin on the viability of PC12 cells was tested by MTT assay (*n* = 6) **(A)** and live/dead cell staining assay. Bar = 200μm **(B)**. **(C)** The ratio of living cells was counted in different groups (*n* = 4). **(D)** TEM images of mitochondrial morphology were obtained and compared between different groups. Black arrows indicate normal mitochondria, and red arrows indicate shrunken mitochondria with cristae broken or disappeared. Bar = 5μm. **(E)** The fluorescence intensity of DHE represents the ROS concentration in different groups. Bar = 200μm. **(F)** Relative fluorescence intensity statistics of the ROS concentration in different groups (*n* = 4). **(G)** The expression of GPX4 was analyzed using immunofluorescence staining. Bar = 200μm **(H)** Relative fluorescence intensity statistics of the expression of GPX4 in different groups (*n* = 4). The experiments were repeated at least three times with three replicates and data were expressed as mean ± SD. ^##^ *p* < 0.01 compared with the control group and ^*^ *p* < 0.05 and **p < 0.01 compared with the RSL3-treated group.

The effect of baicalin on PC12 cells treated with RSL3 was analyzed to further conform whether baicalin exhibited its neuroprotective role through inhibiting ferroptosis. GPX4 is an antioxidant enzyme, which promotes the reduction of lipid peroxides against ferroptosis. Inhibition of GPX4 indeed induces ROS accumulation and subsequent ferroptosis (Friedmann Angeli et al., 2014). RSL3 is a ferroptosis inducer which suppresses GPX4 activity (Sui et al., 2018). In our study, the results of MTT assay and live/dead cell staining assay showed that RSL3 reduced the cell viability, while baicalin dramatically increased the survival rates of PC12 cells treated with RSL3 dose-dependently (Figures 3A–C). The morphological features of ferroptosis were mainly characterized by reduced sizes of mitochondria, membrane thickening, mites disappearing and rupture of the mitochondria (Battaglia et al., 2020). In our study, we observed the morphological features of mitochondria in PC12 cells under a TEM. We found shrunken mitochondria with cristae broken or disappeared in PC12 cells treated with RSL3, while baicalin significantly attenuated the morphological damage in the mitochondria (Figure 3D). Moreover, baicalin significantly reduced the excessive production of ROS induced by RSL3 (Figures 3E,F). Furthermore, baicalin remarkably increased the expression of GPX4 in a dose-dependent manner (Figures 3G,H). This was a clear indication that baicalin effectively prevented the PC12 cells against RSL3-induced ferroptosis.

### Baicalin Inhibited Erastin-Induced Ferroptosis in PC12 Cells

**FIGURE 4:**
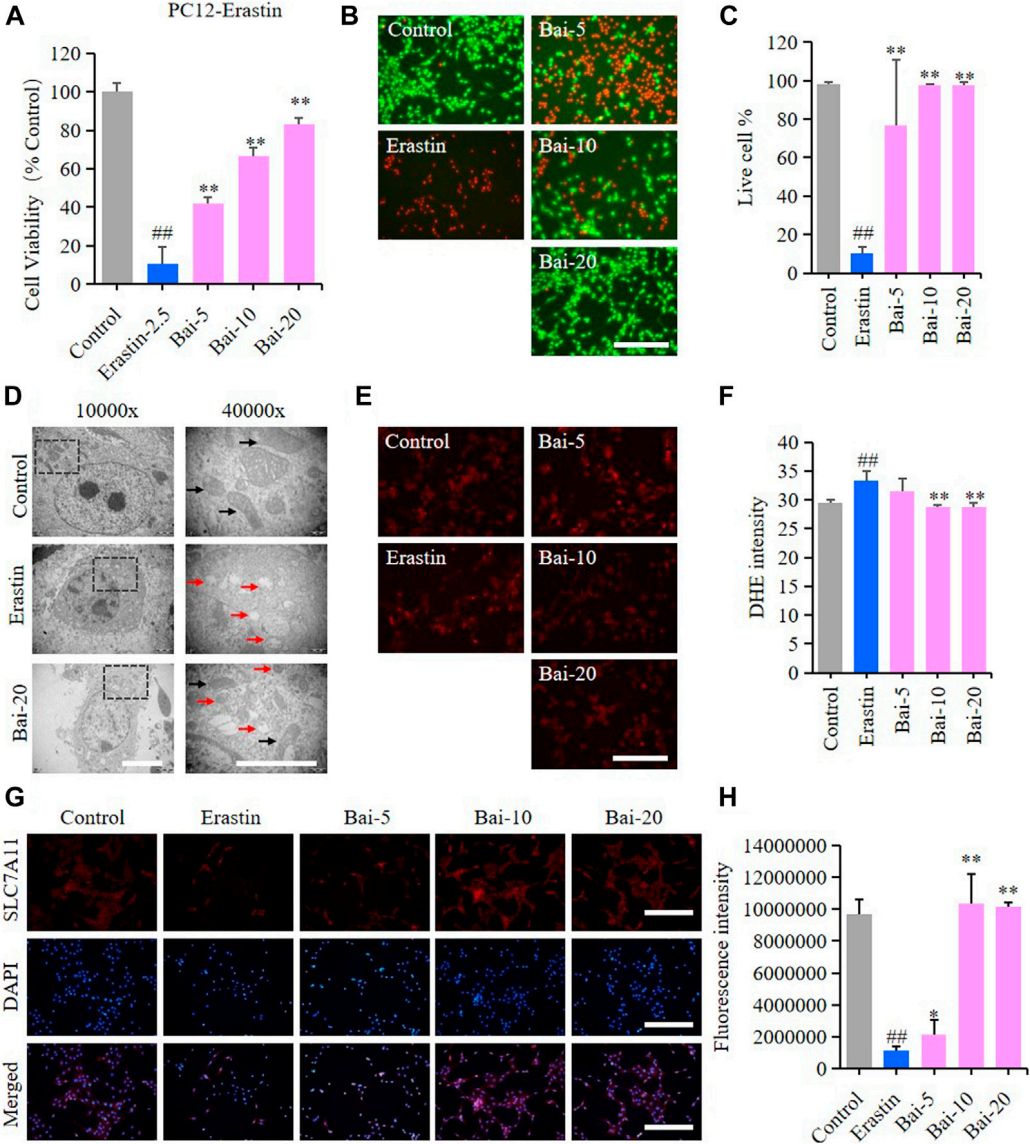
Effect of baicalin on erastin-induced ferroptosis in PC12 cells. PC12 cells were cultured in 96-well plates, treated with erastin (2.5μM) and corresponding concentrations of baicalin (0, 5, 10 and 20μM) for 24h. Effect of baicalin on the viability of PC12 cells was tested by MTT assay (*n* = 6) **(A)** and live/dead cell staining assay. Bar = 200μm **(B)**. **(C)** The ratio of living cells was counted in different groups (*n* = 4). **(D)** TEM images of mitochondrial morphology were obtained and compared between different groups. Black arrows indicate normal mitochondria, and red arrows indicate shrunken mitochondria with cristae broken or disappeared. Bar = 5μm. **(E)** The fluorescence intensity of DHE represents the ROS concentration in different groups. Bar = 200μm **(F)** Relative fluorescence intensity statistics of the ROS concentration in different groups (*n* = 4) **(G)** The expression of SLC7A11 was analyzed using immunofluorescence staining. Bar = 200μm **(H)** Relative fluorescence intensity statistics of the expression of SLC7A11 in different groups (*n* = 4). The experiments were repeated at least three times with three replicates and data were expressed as mean ± SD. ^##^ *p* < 0.01 compared with the control group and *p < 0.05 and **p < 0.01 compared with the erastin-treated group.

Erastin can cause ferroptosis through inhibiting the activity of System Xc−, which is an antiporter responsible for the cellular uptake of cystine in exchange for intracellular glutamate (Yu et al., 2019). In our study, erastin decreased the survival rate of PC12 cells, while baicalin significantly increased the cell viability of PC12 cells treated with erastin (Figures 4A–C). Furthermore, baicalin significantly reversed the morphological damage in the mitochondria of PC12 cells treated with erastin (Figure 4D) and decreased the erastin-induced intracellular ROS accumulation (Figures 4E,F). Importantly, baicalin enhanced the expression level of SLC7A11 (Figures 4G,H), which indicated that baicalin could prevent ferroptosis through enhancing the activity of System Xc−.

### Baicalin Inhibited Ferroptosis in Primary Cortical Neurons

**FIGURE 5:**
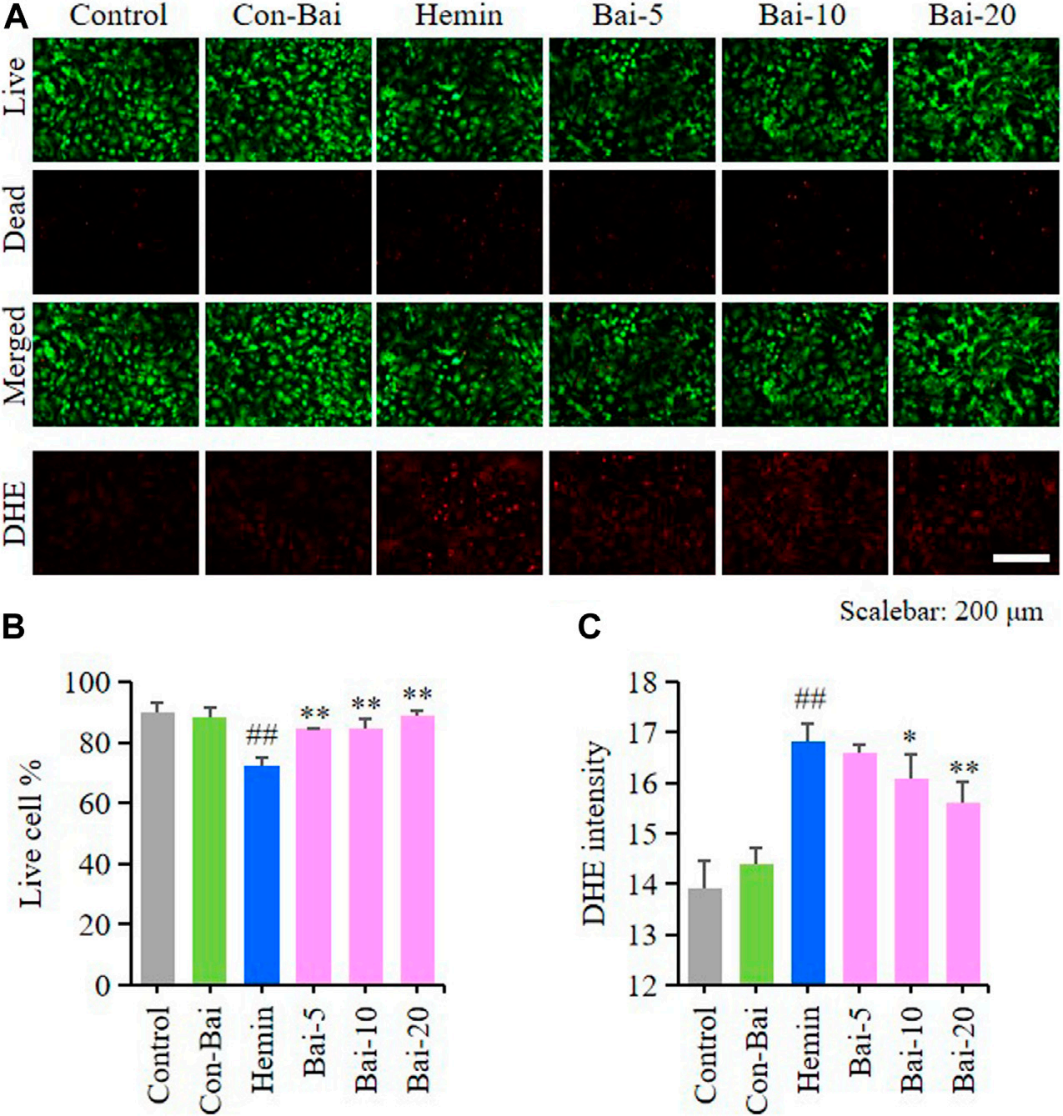
Baicalin showed anti-ferroptosis effect on hemin-treated PCN **(A)** Representative pictures of live/dead cell staining assay and DHE assay in hemin-induced PCN treated with baicalin. Bar = 200μm **(B)** The ratio of living cells was counted in different groups (*n* = 4) **(C)** Relative fluorescence intensity statistics of the ROS concentration in different groups (*n* = 4). The experiments were repeated at least three times with three replicates and data were expressed as mean ± SD. ^##^ *p* < 0.01 compared with the control group and *p < 0.05 and **p < 0.01 compared with the hemin-treated group.

**FIGURE 6:**
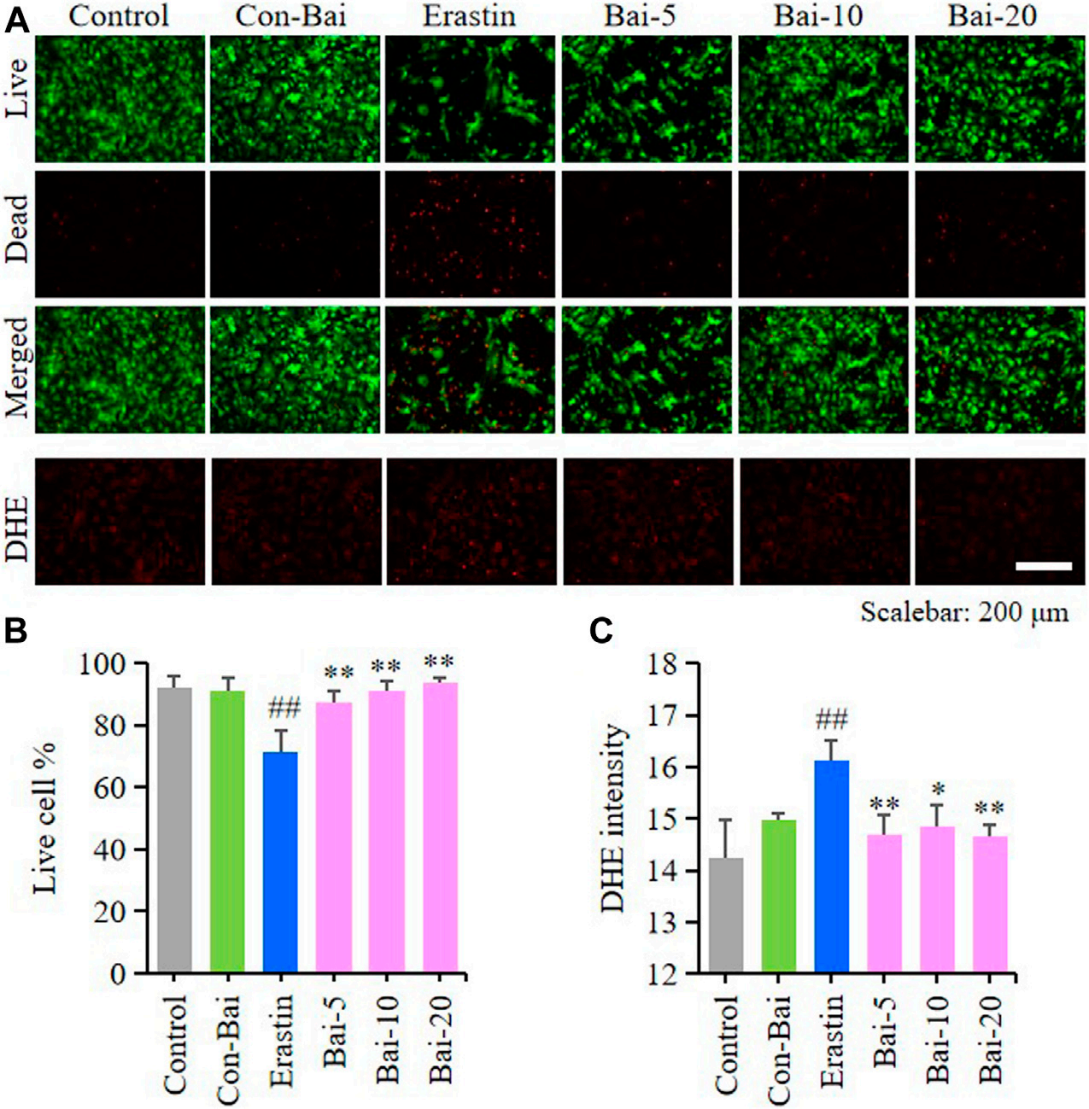
Baicalin showed anti-ferroptosis effect on erastin-treated PCN **(A)** Representative pictures of live/dead cell staining assay and DHE assay in erastin-induced PCN treated with baicalin. Bar = 200μm **(B)** The ratio of living cells was counted in different groups (*n* = 4) **(C)** Relative fluorescence intensity statistics of the ROS concentration in different groups (*n* = 4). The experiments were repeated at least three times with three replicates and data were expressed as mean ± SD. ^##^ *p* < 0.01 compared with the control group and *p < 0.05 and **p < 0.01 compared with the erastin-treated group.

To investigate whether baicalin exist anti-ferroptosis effect on PCN, we performed live/dead cell staining assay and ROS detection in hemin-induced PCN and erastin-induced PCN treated with baicalin. The results showed that compared with the control, hemin caused significant cell death in PCN, while baicalin treatment remarkably increased the cell survival rate (Figures 5A,B). Moreover, hemin enhanced ROS accumulation in PCN, while baicalin administration effectively reduced the level of ROS (Figures 5A,C). On the other hand, the results of live/dead cell staining assay showed that baicalin markedly inhibited the cell death caused by erastin in PCN (Figures 6A,B). Furthermore, baicalin significantly suppressed erastin-induced ROS generation in PCN (Figures 6A,C). These results strongly suggested that baicalin showed anti-ferroptosis effect on PCN.

### Baicalin Alleviated Motor Deficits in Intracerebral Hemorrhage Model Mice

**FIGURE 7:**
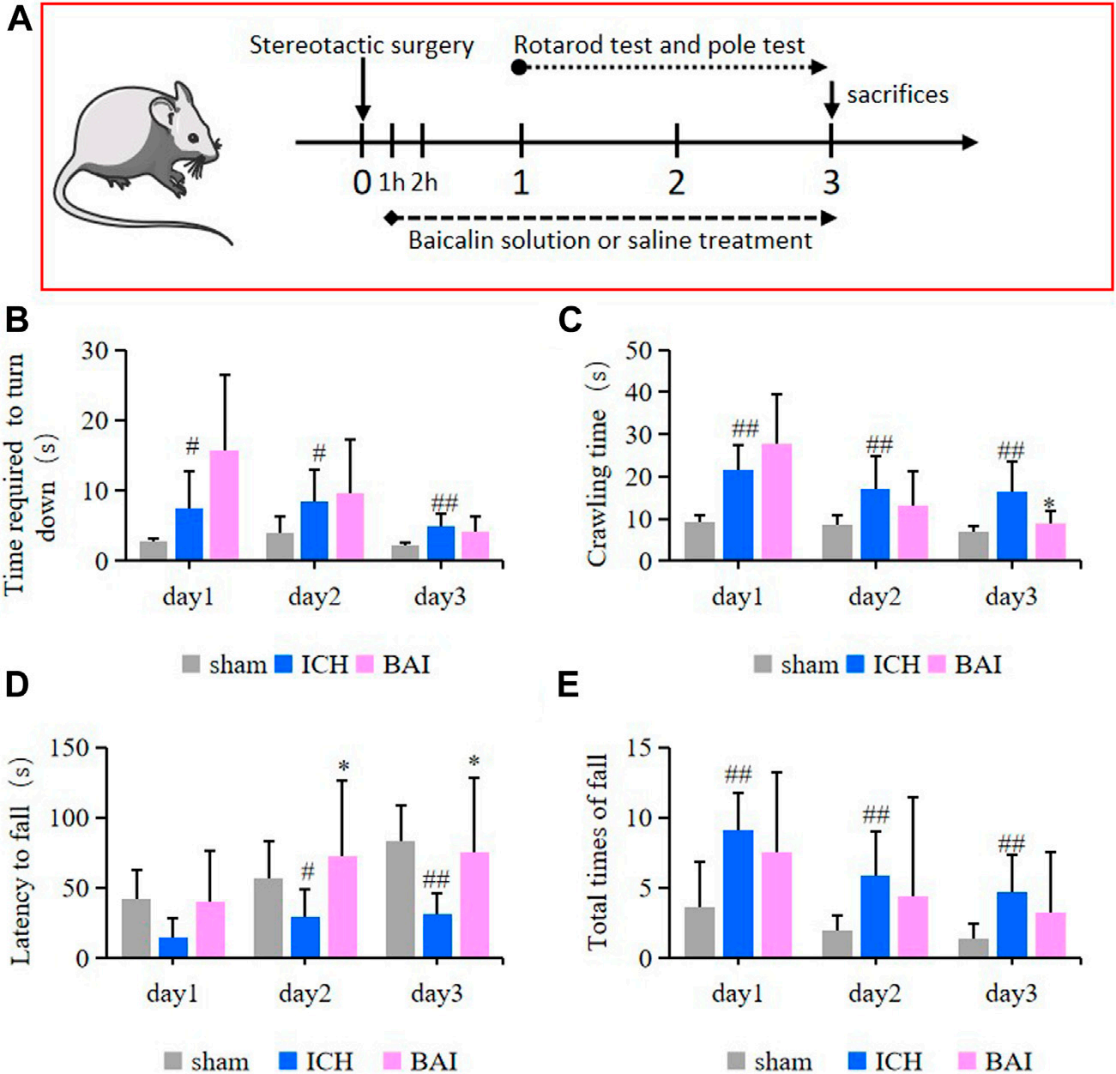
Effect of baicalin on motor deficits in ICH model mice (*n* = 10–12). **(A)** The animal experimental flowchart. The time the mice took to turn downwards **(B)** and the crawling time **(C)** in the pole test were shown. The latency to fall **(D)** and total times of fall **(E)** in the rotarod test were shown. Experimental values were expressed as means ± SD. ^#^ *p* < 0.05 and ^##^ *p* < 0.01 compared with the sham operation group and *p < 0.05 compared with the ICH group.

In this study, the survival of animals was greater than 90%. The efficacy of baicalin in alleviating motor impairment of ICH model mice was evaluated using the pole test and rotarod test. An animal experimental flowchart was displayed in Figure 7A. The results of pole test revealed that compared with ICH model group, the time mice took to turn downwards after placing shortened gradually over time in the Bai group (Figure 7B). In addition, the ICH model mice had a longer crawling time compared to that of mice in the control group, while baicalin reduced the crawling time on day 3 after Type IV collagenase injection (Figure 7C). On the other hand, in the rotarod test, the ICH model mice had a significant decrease in latency to fall compared to those in the Sham group, while baicalin significantly alleviated the disfunction (Figure 7D). Moreover, baicalin reduced the total times of fall in the rotarod test (Figure 7E). The results strongly suggested that baicalin remarkably ameliorated the motor disability in ICH model mice.

### Baicalin Alleviated the Brain Injury in Intracerebral Hemorrhage Model Mice

**FIGURE 8:**
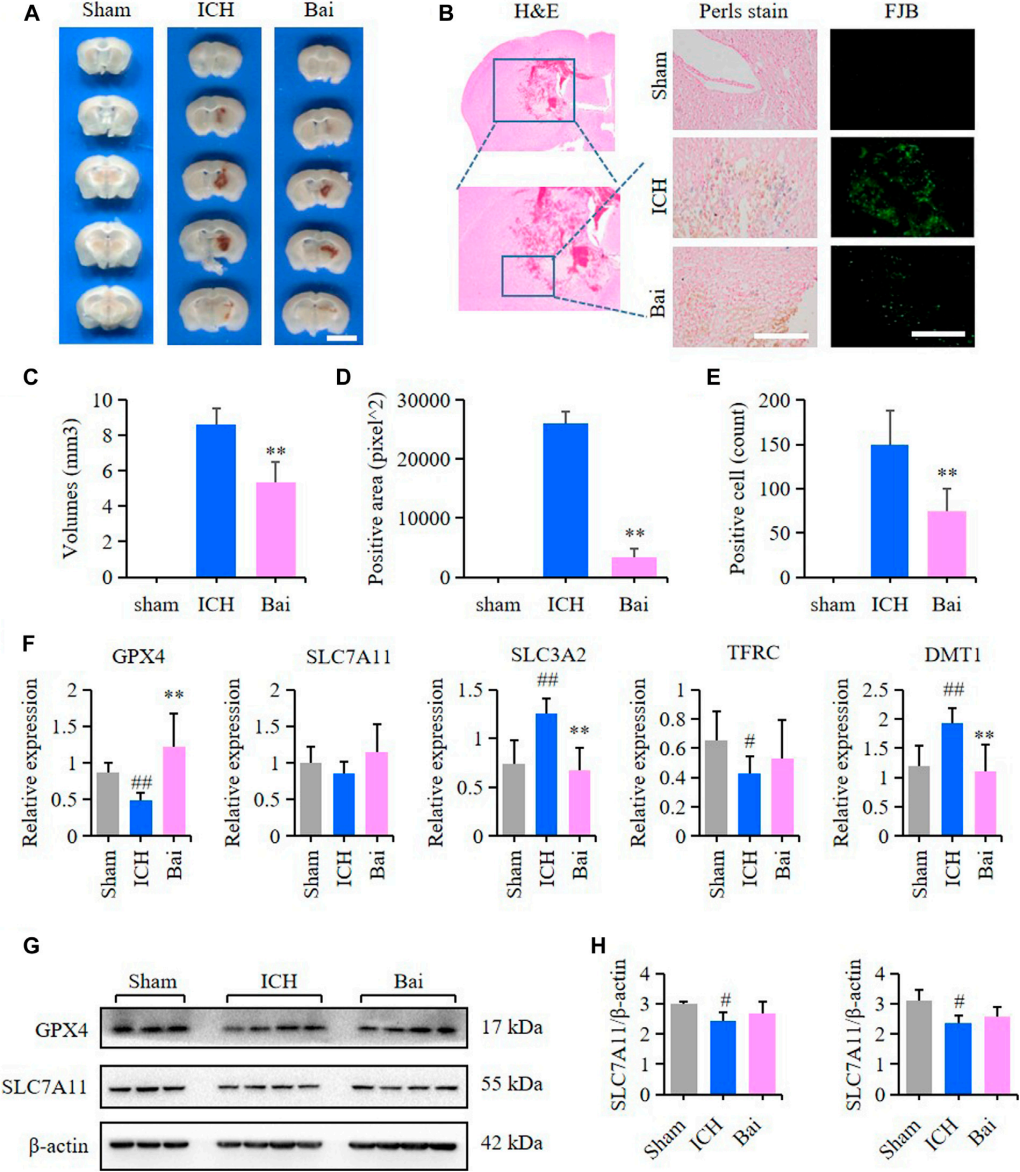
Effect of baicalin on brain injury in ICH model mice. **(A)** Representative pictures of the hemorrhagic lesion in the mice of different groups. Bar = 0.5cm. **(B)** Representative images of Prussian blue staining and FJB staining of brain slices in the mice of different groups. Bar = 200μm. **(C)** Hematoma volume quantitative data of different groups (*n* = 6) **(D)** Relative statistics of Prussian blue labeling density in different groups (*n* = 4). **(E)** Relative fluorescence intensity statistics of FJB staining in different groups (*n* = 4). **(F)** The expression of GPX4, SLC7A11, SLC3A2, TFRC and SLC11A2 (DMT1) in the perihematoma brain tissues of mice were detected by RT-qPCR (*n* = 6) **(G)** The expression of GPX4 and SLC7A11 in the perihematoma brain tissues of ICH model mice were detected by western blot **(H)** Quantification data of western blot in different groups (*n* = 4). Experimental values were expressed as means ± SD. ^#^ *p* < 0.05 and ^##^ *p* < 0.01 compared with the sham operation group and **p < 0.01 compared with the ICH group.

The efficacy of baicalin in reducing hematoma volume was assessed by scanning of brain slices. As shown in Figures 8A,C, the pathological observation of brain tissues showed significant hematoma in the Mod group. In contrast, baicalin remarkably diminished the volume of hematoma after ICH injury. The localization of ferric iron in the perihematoma brain tissues was detected by Prussian blue staining. The deposition of ferric iron in the perihematoma brain tissues was obviously increased after ICH injury. However, baicalin significantly reversed this effect (Figures 8B,D). These findings suggested that baicalin was effective in attenuating iron deposition in the perihematoma brain tissues of ICH model mice. The results of FJB staining revealed that ICH injury induced remarkable neuronal degradation in the perihematoma brain tissues of ICH model mice. However, these damages were attenuated by baicalin treatment (Figures 8B,E). These results indicated that baicalin could obviously ameliorate the neuronal degradation caused by ICH injury. The mRNA expression levels of GPX4, SLC7A11, solute carrier family three membrane 2 (SLC3A2), transferrin receptor (TFRC) and solute carrier family 11 membrane 2 (SLC11A2, DMT1) in the perihematoma brain tissues were measured using RT-qPCR to detect the effect of baicalin on ferroptosis *in vivo*. Baicalin significantly increased the expression levels of anti-lipid oxidation mRNA GPX4 and SLC7A11 and inhibited the expression of iron transport mRNA SLC11A2 (Figure 8F) *in vivo*. Moreover, the results of western blot revealed that baicalin treatment could enhanced the expression of GPX4 and SLC7A11 to some degree (Figures 8G,H).

### Effect of Baicalin on the Structures and Functions of Liver and Kidney

**FIGURE 9:**
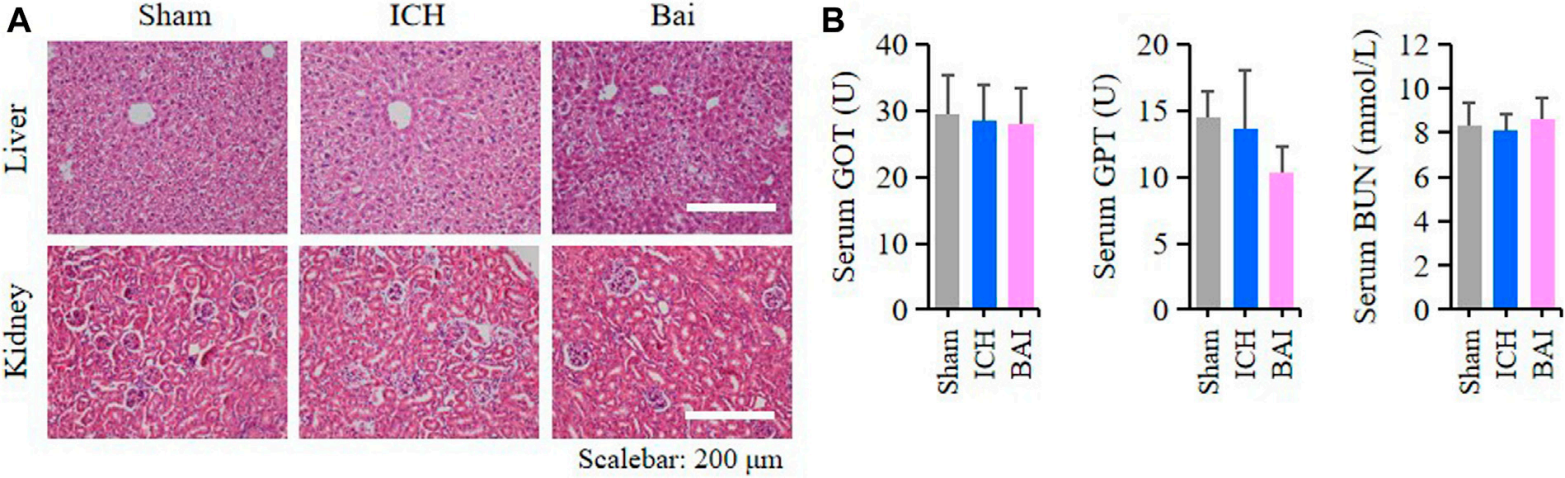
Baicalin existed no obvious toxicity towards the liver and kidney. **(A)** Representative pictures of HE staining of liver and kidney structures in different groups (*n* = 4). Bar = 200μm. **(B)** Bar charts of serum levels of GOT, GPT and BUN in different groups (*n* = 6).

The results HE staining showed that baicalin did not affect the structures of liver and kidney tissues in the mice of each group (Figure 9A). Moreover, baicalin showed no impact on the serum levels of GOT, GPT and BUN in the mice (Figure 9B). These results indicated that baicalin existed no obvious toxicity towards the liver and kidney.

## Discussion

Our study explored the effect and mechanisms of baicalin on ferroptosis caused by ICH injury both *in vitro* and *in vivo*. We found that baicalin significantly inhibited ferroptosis in PC12 cells treated with hemin, RSL3 and erastin. Importantly, baicalin showed anti-ferroptosis effect on PCN. Further *in vivo* experiments revealed that baicalin remarkably alleviated motor deficits and brain injury through inhibiting ferroptosis in ICH model mice. Additionally, baicalin existed no obvious toxicity towards the liver and kidney of mice. These results strongly suggested that baicalin could be an effective drug candidate for ICH.

Lyzed erythrocytes release hemoglobin after ICH injury. Their degradation product hemin is further resolved into iron ions by HO-1 (Wei et al., 2017). Then excess iron ions lead to free radical production by Fenton reaction thereby causing oxidative damage to lipids, proteins and DNA (Pantopoulos et al., 2012; Fanzani and Poli, 2017). The hemin-treated PC12 cell model is a classical experimental model to mimic the neuronal damage process of ICH in human (Wang et al., 2017). Our study showed that hemin significantly decreased the cell viability of PC12 cells. Moreover, identified ferroptotic hallmarks were identified in the hemin-treated PC12 cells. Consistent with our findings, a resent research reported that the infusion of hemoglobin and its degradation products caused brain injury in rats by aggravating iron deposition (Wang et al., 2013). Collectively, these findings highlight the potential therapeutic value of targeting the ferroptosis process to cure ICH injury.

Ferroptosis is defined as a new iron and lipid peroxidation-dependent form of programmed cell death. It was first described in RAS mutant cancer cells in 2012 (Dixon et al., 2012). Morphologically, ferroptosis has typical necrotic features, such as dysmorphic small mitochondria with decreased crista, condensed membrane and ruptured outer mitochondrial membrane rupture (Cao and Dixon, 2016; Gao et al., 2019; Tadokoro et al., 2020). Inactivating the major protective mechanism of membranes against peroxidation damage is the canonical pathway of ferroptosis (Latunde-Dada, 2017). System Xc− is an indispensable player in the regulation of lipid peroxidation. It is a heterodimeric cystine/glutamate antiporter composed of SLC7A11 and SLC3A2, which plays a vital role in maintaining the redox balance of antioxidant GSH synthesis (Yu et al., 2017). GPX4 is an important cofactor that detoxifies hydroperoxides in complex lipids by using GSH. Inhibition of system Xc− or GPX4 can cause ferroptosis through reducing GSH level, and subsequently lead to the accumulation of ROS that finally result in lipid peroxidation (Doll et al., 2017; Stockwell et al., 2017). Brain tissues contain high-rich phospholipids which are more susceptible to lipid peroxidation damage (Choi et al., 2018). Importantly, increasing evidence suggests that ferroptosis could be the leading factor to induce ICH secondary injury. The ferroptosis inhibitors have become increasingly popular in ICH therapy recently (Cao et al., 2021).

Baicalin is a main active ingredient of Chinese herbal medicine Scutellaria baicalensis. An *in vivo* study measuring baicalin concentration in rat blood and brain demonstrated that baicalin can cross the BBB and distribute into the cerebrospinal fluid quickly and reach its peak concentration of 344g/L about 30min after the i. v. administration of 24mg/kg (Huang et al., 2008). Pharmacokinetic studies showed that the distribution of baicalin into brain was a subsequent process and baicalin tended to accumulate in the striatum, thalamus and hippocampus with the exhibition of large area (Zhang et al., 2006). Moreover, it was reported that the distribution half-life and elimination half-life of baicalin in normal rats was 0.8868 and 26.0968min respectively, while corresponding parameters were 2.084min, 34.4998min in cerebral ischemia-reperfusion rats (Li et al., 2018). Additionally, the middle cerebral artery occlusion rats showed better, quicker absorption of baicalin than sham-operated rats. The data indicated that in the pathologic condition, baicalin might have a better absorption effect, and it proves the rationality of using it in the cerebrovascular disease, which would improve the therapeutic efficacy (Zeng et al., 2010; Liang et al., 2017). In our study, we found that baicalin could significantly increase the viability of PC12 cells treated with hemin.

These beneficial properties impel us to further determine the protective mechanism of baicalin in ICH injury. Lipid peroxidation and iron accumulation are essential elements for the execution of ferroptosis (Yang and Stockwell, 2016). Ferroptosis occurs when GPX4-catalyzed reduction of lipid hydroperoxides is not enough to inhibit the iron-induced generation of lipid radicals (Imai et al., 2017). As such, we investigated the expression levels of GPX4 and SLC7A11 in PC12 cells treated with hemin. Baicalin enhanced the expression of GPX4 and SLC7A11, suggesting the restoration of System Xc− and GPX4 activity in PC12 cells. These findings further revealed that baicalin was effective in preventing the development of ferroptosis in PC12 cells treated with hemin. Importantly, baicalin also significantly inhibited ferroptosis induced by RSL3 and erastin, which further confirmed the cytoprotective effect of baicalin against ferroptosis.

Next, we investigated the effect of baicalin on motor deficits and neuropathological changes *in vivo*. Type IV collagenase induced mouse model is often employed to mimic the pathological changes of ICH in previous studies (Krafft et al., 2012). In this study, we established a ICH animal model by injecting mice with Type IV collagenase. In the *in vivo* experiment, we found baicalin treatment alleviated motor disorders and brain injuries including hematoma, iron deposition and neural degeneration in the perihematoma brain tissues caused by ICH. Furthermore, baicalin significantly increased the mRNA expression of GPX4 and SLC7A11 in the perihematoma brain tissues of ICH model mice. The intracellular transport of iron ions depends on transferrin (Gao et al., 2015). Iron combines with transferrin and then enters the cells via TFRC. Divalent metal ion transporter SLC11A2 also participates in the iron transport. TFRC and SLC11A2 genes both regulate the intracellular iron deposition (Kleven et al., 2018). Therefore, we measured the levels of TFRC and SLC11A2 (DMT1) mRNA with the purpose to confirm whether baicalin could regulate the intracellular transport of iron ions. We found that baicalin obviously decreased the expression level of SLC11A2 (DMT1), which indicated that baicalin could inhibit iron transport in the perihematoma brain tissues. Nevertheless, baicalin showed no significant effect on TFRC expression. Collectively, these results revealed that baicalin mitigated ICH-induced brain injury in ICH model mice through preventing ferroptosis.

**FIGURE 10:**
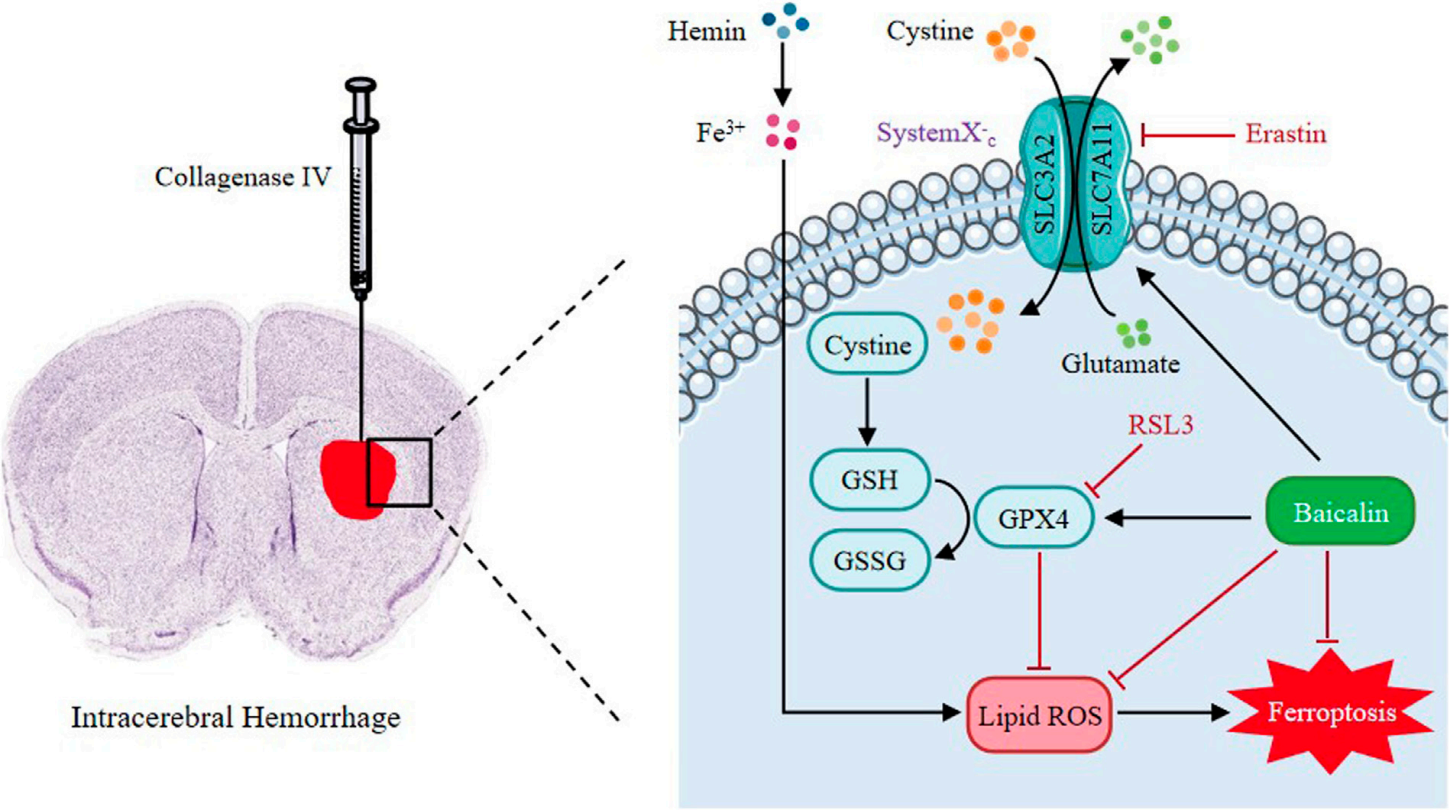
Proposed mechanistic model of baicalin protects against hemin-induced ferroptosis in ICH. Our findings demonstrated that baicalin effectively enhances the expression levels of GPX4 and SLC7A11, and inhibits the accumulation of lipid ROS and eventually protects against hemin-induced ferroptosis and ICH-induced brain injury.

Nevertheless, our study was limited by several factors. Our present study only explored baicalin’s role in lipid peroxidation and intracellular transport of iron in inhibiting ferroptosis, while it could also inhibit ferroptosis through a variety of other ways. In addition, the study only evaluated the (Figure 10) anti-ferroptosis properties of baicalin without further investigating its effects on other mechanisms. We did not evaluate the long-term benefit of baicalin on ICH. Cognizant to this, further studies on the mechanisms of baicalin in inhibiting ferroptosis after ICH injury should be conducted to shed more light on the subject.

## Conclusion

In conclusion, all these findings revealed that ferroptosis is a key pathological feature of ICH. Baicalin can inhibit the development of ferroptosis in ICH. Baicalin is a potential therapeutic drug for ICH treatment.

## Data Availability

The original contributions presented in the study are included in the article/Supplementary material, further inquiries can be directed to the corresponding authors.
